# Solid state interdigitated Sb_2_S_3_ based TiO_2_ nanotube solar cells†

**DOI:** 10.1039/d0ra04123h

**Published:** 2020-07-28

**Authors:** Pascal Büttner, Dirk Döhler, Sofia Korenko, Sebastian Möhrlein, Sebastian Bochmann, Nicolas Vogel, Ignacio Mínguez-Bacho, Julien Bachmann

**Affiliations:** Chemistry of Thin Film Materials, Department of Chemistry and Pharmacy, IZNF, Friedrich-Alexander University of Erlangen-Nürnberg Cauerstr. 3 91058 Erlangen Germany ignacio.minguez@fau.de julien.bachmann@fau.de; Department of Chemical and Biological Engineering, Friedrich-Alexander University of Erlangen-Nürnberg HaberstraÃ§e 9a 91058 Erlangen Germany; Institute of Chemistry, Saint-Petersburg State University Universitetskii pr. 26 198504 St. Petersburg Russia

## Abstract

TiO_2_ nanotubes generated by anodization of metallic titanium sputter-coated on indium tin oxide (ITO) substrates are used as a conductive scaffold for all solid-state Sb_2_S_3_-sensitized extremely thin absorber (ETA) solar cells. A blocking layer of TiO_2_ placed between Ti and ITO in combination with optimized Ti deposition and anodization conditions enables the formation of crack-free layers of straight, cylindrical TiO_2_ nanotubes of tunable length and diameter. ALD (atomic layer deposition) is subsequently used to coat this substrate conformally with a highly pure Sb_2_S_3_ light absorber layer under an inert atmosphere. The high absorption coefficient of Sb_2_S_3_ as compared to molecular dyes allows for the utilization of very short nanotubes, which facilitates the infiltration of the organic hole transport material and formation of a p–i–n heterojunction in an interdigitated and tunable geometry. We investigate the influence of nanotube length and of the absorber thickness to enhance the photocurrent value to twice that of planar reference structures.

## Abstract 2

TiO_2_ nanotubes generated by anodization of metallic titanium sputter-coated on indium tin oxide (ITO) substrates are used as a conductive scaffold for all-solid-state Sb_2_S_3_-sensitized extremely thin absorber (ETA) solar cells.

## Graphical abstract

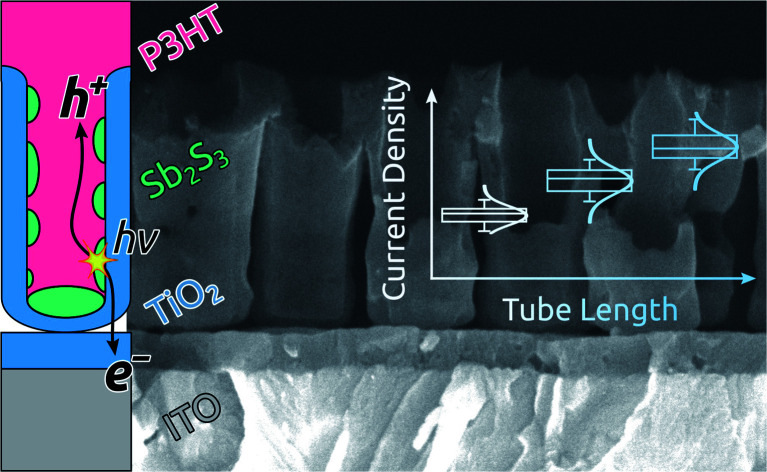

## 1 Introduction

Structuring semiconductor interfaces on the nanoscale holds significant potential in the field of emerging photovoltaics, enabling the utilization of non-toxic materials composed of abundant elements that would not be applicable otherwise. The geometry of the interface determines the effectiveness of light absorption, the transport distances of charge carriers and their chance of recombination.^1^ In the typical mesoporous TiO_2_ layer, however, a systematic study of how geometric parameters can be exploited to affect photovoltaic performance is difficult due to the absence of independent experimental handles on transport distances and absorption cross-section.^2^ The interdigitated junction geometry has been hailed in this context as the ‘holy grail’, since a parallel array of ordered, nanocylindrical junctions would allow for a systematic study of these geometric effects.^3^ Here, we establish a preparative method for such an interdigitated heterojunction in all solid-state solar cells based on titanium dioxide nanotubes (TiO_2_ NTs) and antimony sulfide (Sb_2_S_3_, stibnite), and we demonstrate how geometric parameters affect the photovoltaic performance parameters. Table S1 in the ESI† summarizes the state of the art concerning Sb_2_S_3_-based solar cells in which an interdigitated, coaxial geometry of the semiconductor heterojunction has been achieved.

Titanium dioxide is not only a wide-bandgap, n-type semiconductor but also a solid of outstanding stability, high abundance and low toxicity.^4^ Electrochemical anodization of titanium foils or thin films to TiO_2_ NTs represents an inexpensive and simple method for the formation of geometrically well-defined TiO_2_ nanostructures with high scalability.^2^ The geometry of TiO_2_ NTs can easily be adjusted by varying the anodization conditions, such as applied potential and electrolyte composition, which enables tailoring the structure exactly to their respective applications.^2^ The resulting structures provide a large surface area in combination with short and direct carrier pathways for various electronic applications, such as batteries,^5,6^ catalysis,^7^ water splitting^8,9^ and emerging photovoltaics.^2^ In the photovoltaics (PV) field, TiO_2_ NTs have been used to make dye-sensitized solar cells (DSSCs)^10,11^ as well as perovskite^12,13^ and polymer bulk heterojunction cells,^14^ where their quasi-1D structure and the adjustability of their geometry has allowed for a much more thorough fine-tuning than the commonly used mesoporous device architecture. Beyond these three classes of PV devices, however, the potential of TiO_2_ NT substrates has not been demonstrated. Mostly, a liquid electrolyte is used to transport holes and allows for addressing the full surface of the geometrically challenging, deep pores.^2^

The strategy that we propose here relies on an inorganic, solid-state light absorber layer with higher absorption cross-section than molecular dyes used in DSSCs, which allows for shorter tubes to be used. This is the so-called ‘extremely thin absorber’ (ETA) principle.^15^ Filling these short tubes with a polymer hole transport material (HTM) such as poly-3-hexylthiophene (P3HT) instead of a liquid electrolyte allows for the formation of an interdigitated structure with short carrier pathways. It eliminates the major concern of electrolyte leakage in traditional DSSCs and it reduces the series resistance.^16^ This strategy, however, relies on anodic TiO_2_ NTs generated on a transparent conducting substrate directly. Such structures have not been involved in ETA cells so far.

As the light absorber solids, chalcogenide semiconductors such as indium sulfide (In_2_S_3_),^17^ copper sulfide (Cu_*x*_S),^18^ and copper zinc tin sulfide (Cu_2_ZnSnS_4_, CZTS)^19,20^ have been employed with particular success. We choose to use antimony sulfide instead, since this material combines the advantages of relative abundance, low toxicity and high stability.^21,22^ The bandgap of 1.7 eV and high absorption coefficient of 1.8 × 10^5^ cm^−1^ at 450 nm render Sb_2_S_3_ highly promising as an absorber material for emerging photovoltaics.^23^ Although Sb_2_S_3_ has successfully been employed in thin film solar cells,^22,24–28^ to date the record efficiency for this material was achieved in a nanostructured, but disordered, mesoporous TiO_2_ based configuration.^29^

Sb_2_S_3_ is usually deposited from solution based methods, such as chemical bath deposition and spin coating.^30–33^ However, the tendency of Sb_2_S_3_ to form deep traps upon oxygen incorporation makes it mandatory to use water- and oxygen-free conditions.^25^ In this context, atomic layer deposition (ALD) represents the ideal method for the deposition of thin Sb_2_S_3_ absorber layers under inert conditions, since it is inherently capable of coating on non-planar substrates in a conformal manner.^24,25,34–36^

In this study, we present TiO_2_ NT-based solid-state heterojunction solar cells with ALD-grown Sb_2_S_3_ as the absorber material. We explore the influence of NT length and Sb_2_S_3_ loading as compared to planar device architecture. This system is the first Sb_2_S_3_-sensitized solar cell on a nanotubular platform. The goal is to establish a model system for mesoporous cells, featuring direct and straight transport paths of the photoseparated charge carriers from the junction to the electrode. In this highly controlled geometry, one can experimentally thin down the light absorbing layer and optimize the total light absorption accordingly by elongating the pores as needed. This kind of cell offers two experimental parameters fully decoupled from each other (the pore length and absorber layer thickness) and represents the missing link between the classical planar and mesoporous cell architectures.

## 2 Experimental section

### 2.1 Sample preparation

ITO coated glass substrates purchased from Techinstro with a sheet resistance of 10 Ω sq^−1^ are first cleaned by sonication in Hellmanex III (detergent solution, 1%), acetone and isopropanol for 5 min each and subjected to UV–ozone cleaning for 30 min right before use. A blocking layer of 45 nm amorphous TiO_2_ (TiO_2_ target, 99.99%) is deposited by radio frequency magnetron sputtering (CRC 622 model, Torr International, Inc.) at a working pressure of 0.3 Pa with a power density of 2.5 W cm^−2^, resulting in a deposition rate of 0.1 Å s^−1^. The working gas is argon at a flow rate of 5 sccm. The base pressure is 1 × 10^−4^ Pa. Thereafter, the coated samples are again cleaned with UV–ozone for 30 min immediately before deposition of the Ti metal films. 170, 340 and 510 nm of Ti thin films are sputtered from a Ti target (99.9%). HQ Ti films are deposited at a working pressure of 0.3 Pa with a power density of 3.3 W cm^−2^ and a Ar flow of 5 sccm, whereas LQ Ti films are sputtered at 0.5 Pa, 2.7 W cm^−2^ and a Ar flow of 10 sccm. The resulting deposition rate is approximately 0.8 Å s^−1^ in both cases. The Ti thin films are anodized in a two-electrode setup in an ethylene glycol based electrolyte containing 0.5% NH_4_F, 3% water and 0.5 wt% H_3_PO_4_ (85%) at 60 V. The endpoint of anodization is reached when the sample turns transparent and the sample is then left in the electrolyte for 20 min without applied potential to widen the inner tube diameter. The samples are then rinsed with ethanol and kept in an ethanol bath over night. After transferring the substrates to a deionized water bath for 1–2 h, the samples are then left to dry in air. Annealing of the as-grown amorphous TiO_2_ NTs is performed at 500 °C for 1 h on a hotplate in ambient atmosphere.

ALD of ZnS and Sb_2_S_3_ is performed in a home-made hot-wall ALD reactor. The reactants are diethylzinc (95%, abcr), tris(dimethylamido)antimony (TDMASb, 99.99%, Sigma Aldrich) and hydrogen sulfide (3% in N_2_, Air Liquide). All precursors are kept at room temperature, except for the TDMASb which was heated to 40 °C due to the lower vapor pressure. The deposition temperature is 150 °C for ZnS and 120 °C for Sb_2_S_3_, respectively. The opening, exposure and pumping times are 0.2, 30 and 40 s in all cases, except for the TDMASb. Here, a longer opening time of 1.5 s is chosen. The Sb_2_S_3_ deposition is performed right after completed ZnS deposition in the same reactor without breaking vacuum. The amorphous as-deposited absorber layer is annealed in a N_2_-filled glovebox on a hotplate at 300 °C for 2 min.

Poly-3-hexylthiophene (P3HT, regioregular, Sigma Aldrich, 15 mg mL^−1^ in chlorobenzene) is spin coated under ambient conditions as hole selective layer at 2000 rpm for 1 min with an acceleration of 500 rpm s^−1^. Due to the limited wetting of the P3HT solution on planar samples, here 50 μL of P3HT are dropped on the substrate at 6000 rpm and kept spinning for 1 min. The P3HT-layer is then dried on at hotplate at 90 °C in an N_2_-filled glovebox. To improve the P3HT–Au contact, an additional layer of poly(3,4-ethylenedioxythiophene) polystyrene sulfonate (PEDOT:PSS, HTL solar, Ossila) is spun at 6000 rpm for 1 min with an acceleration of 6000 rpm s^−1^ and again dried on at hotplate at 90 °C in an N_2_-filled glovebox. Finally, 80 nm of Au are evaporated through a shadow-mask to form an active device area of 0.075 cm^2^.

### 2.2 Characterization

For SEM micrographs a JEOL JSM 6400 equipped with a LaB_6_ cathode and an EDX detector from SAMx or a Gemini 500, Carl Zeiss field-emission instrument was used. The thickness of the as-grown ALD layers was determined by spectroscopic ellipsometry (SENpro, Sentech). XRD diffractograms were recorded on a Bruker D8 Advance with a Cu Kα source and a LynxEye XE T detector. Optical transmission spectra were measured with a DH-2000-L light source and a HR40000 spectrometer. A solar simulator (Newport) equipped with a Xe lamp source was calibrated to AM1.5 (100 mW cm^−2^) with a reference Si solar cell (Newport) for photovoltaic characterization electrical data was recorded using a single-channel Gamry Reference 600 instrument. EQE spectra were measured using a Oriel's QEPVSI-b equipped with a 300 W Xe light source, a monochromator and a lock-in amplifier.

## 3 Results and discussion

### 3.1 Preparation of TiO_2_ NTs by anodization on a transparent conducting oxide (TCO)

**Fig. 1:**
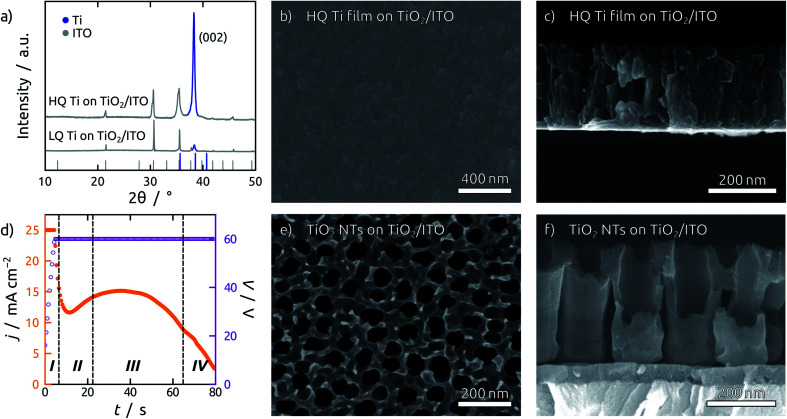
(a) XRD pattern of sputtered Ti thin films on ITO with a working pressure and power density of 0.3 Pa and 3.3 W cm^−2^ (HQ Ti on ITO) and 0.5 Pa and 2.7 W cm^−2^ (LQ Ti on ITO). The powder patterns for In_2_O_3_ (COD ID: 1010341) and Ti (COD ID: 1532765) are indicated in grey and blue, respectively. (b and c) SEM top view (b) and cross section (c) of HQ Ti films on ITO substrates with a sputtered amorphous TiO_2_ blocking layer. (d) Representative current profile during anodization, exhibiting four main stages. Note that the current during anodization is capped at 25 mA cm^−2^. (e and f) SEM top view (e) and cross section (f) of the resulting TiO_2_ NT layers on TiO_2_/ITO after completed anodization and annealing.

The TiO_2_ NT substrates are prepared by direct anodization of Ti films sputter-coated on indium tin oxide (ITO) substrates. The quality of the sputtered Ti films is of prime importance, since smooth and dense films are required for anodization.^37^ During sputter deposition, the quality of the Ti thin film can be enhanced by employing low working pressures and high power densities, which increase the kinetic energy of the Ti atoms impinging on the substrate surface.^38^ Ti atoms with excess kinetic energy are more likely to find a suitable spot in the growing crystal lattice, leading to a superior crystallinity of the Ti film. Fig. 1a compares Ti films grown in different sputtering conditions. High quality (HQ) Ti films are grown at a lower working pressure (0.3 Pa) and higher power density (3.3 W cm^−2^) than the low quality (LQ) Ti films (0.5 Pa and 2.7 W cm^−2^). The X-ray diffraction (XRD) pattern shows a preferred growth in the (001) direction with significantly higher intensity for the HQ film grown. Simultaneously, the so-called ‘atomic peening’ effect helps smooth out the film by resputtering exposed regions during sputter deposition (Fig. 1b, c and S1†).^38^ Additionally, the substrates employed should be as smooth as possible, as the shadow effect during sputtering exacerbates any preexisting roughness. This can be observed in the scanning electron micrographs (SEM) in Fig. S1.† Fluorine doped tin oxide (FTO) is composed of large grains and yields individual, loosely connected Ti pillars (Fig. S2b†), as opposed to the results obtained with ITO (Fig. 1c). The anodization of such LQ Ti layers results in the formation of rather undefined porous structures with major areas of exposed substrate (Fig. S2c†). In contrast to this, anodization of the HQ Ti layers delivers TiO_2_ NT structures consisting of uniform and straight channels connected to each other at their top extremities (Fig. 1e and f).

A second issue of crucial importance for building PV devices of reproducible properties is the necessity to avoid cracks. Even HQ Ti films on planar ITO substrates are affected by the formation of cracks in the last stages of anodization. This obstacle is circumvented by a 45 nm layer of amorphous TiO_2_ sputter-deposited on the ITO substrate before metallic Ti. This blocking layer serves a twofold purpose. First, it avoids over-anodization, as the formation of oxygen bubbles underneath the TiO_2_ NTs after complete consumption of the Ti layer leads to cracking and lift-off (Fig. S3†). Second, even if some cracks were to be formed during anodization, the TiO_2_ blocking layer still prevents direct contact of the subsequent layers to the ITO contact, thereby eliminating short-circuits.

In these optimized conditions, anodization of a Ti thin film proceeds in four major stages, as indicated in a representative current profile during anodization in Fig. 1d.^39^ Stage I shows an exponentially decreasing current as a compact oxide layer is grown. The local breakdown of this oxide layer results in the initialization of NT growth in stage II, which is marked by an increase in current. When NT initialization is completed and the tubes grow at steady state in stage III, the current remains roughly constant, until it starts falling off when the entire thickness of the Ti film has been consumed (stage IV). At this point the film becomes transparent and the anodization stops, blocked by the underlying amorphous TiO_2_ layer.

**Fig. 2:**
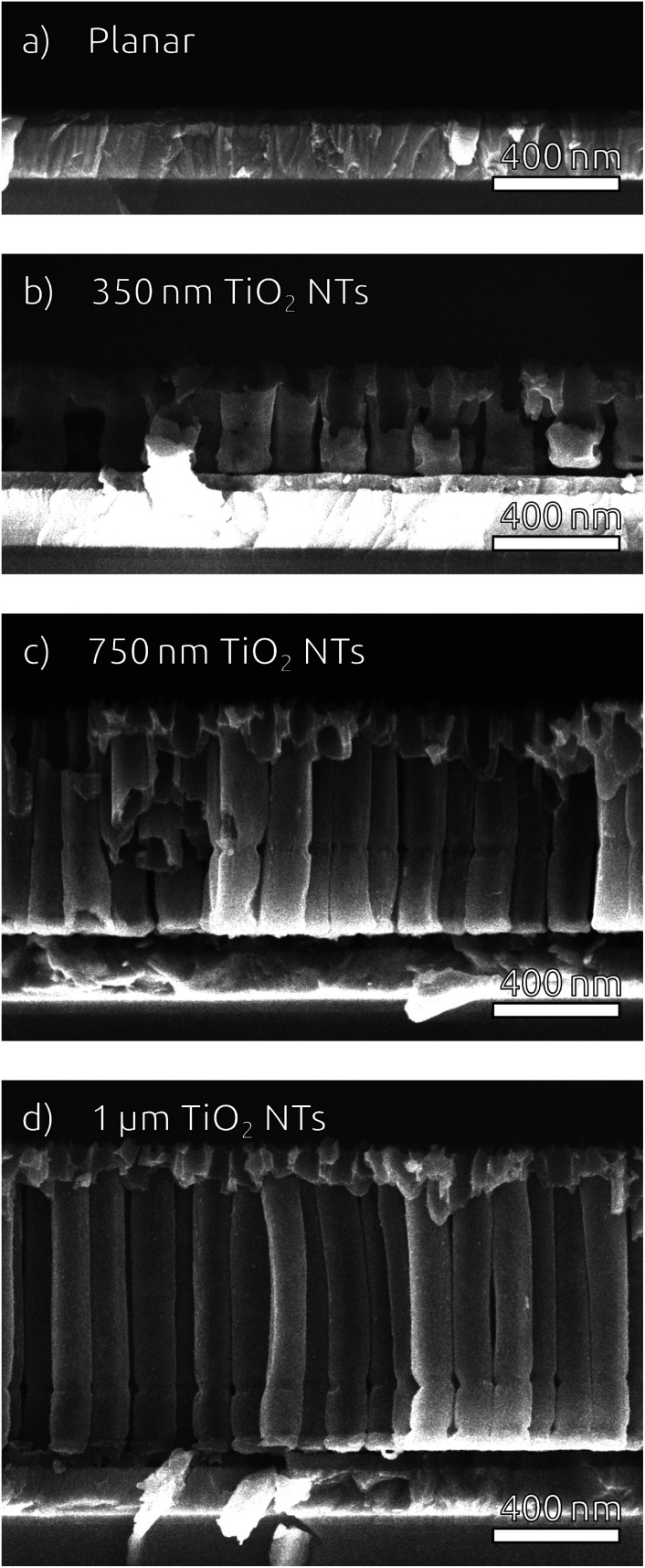
SEM cross-sections of the substrates used for solar cells, featuring anatase TiO_2_ NTs of various lengths on ITO substrates equipped with a sputtered TiO_2_ blocking layer. (a) Planar, (b) 350 nm, (c) 750 nm, (d) 1 μm.

To widen the inner tube diameter, the as-grown TiO_2_ NTs are subsequently left in the electrolyte at open circuit for 20 min (Fig. S4†). The resulting NT films (Fig. 1e and f) feature average inner tube diameters of 80 nm, with a tube length adjustable on the basis of the original Ti thickness. Overall, the anodization procedure causes an expansion by a factor of roughly 2.2 with respect to the sputtered Ti. In the following, we will consider TiO_2_ NT substrates with tube lengths of 350 nm, 750 nm and 1 μm, as well was planar control samples which only possess the compact TiO_2_ blocking layer (Fig. 2). All of them consist of crystalline anatase after annealing at 500 °C.

### 3.2 Atomic layer deposition of Sb_2_S_3_ absorber

**Fig. 3:**
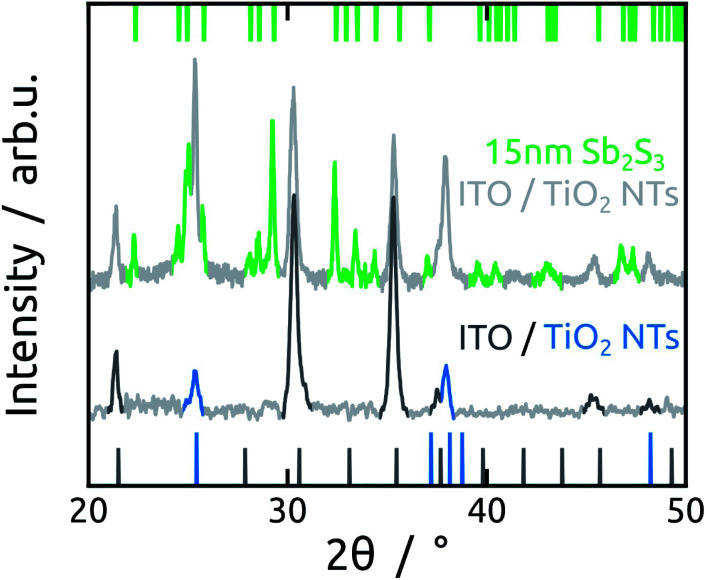
XRD patterns of bare TiO_2_ NTs array on ITO substrate and of TiO_2_ NTs coated with Sb_2_S_3_ (15 nm) on ITO. The expected signals are observed for the following phases: ITO (In_2_O_3_, COD ID 1010341, dark grey), anatase TiO_2_ (COD ID: 1010341, blue) and stibnite Sb_2_S_3_ (COD ID 1011154, green).

In the subsequent steps, ZnS and Sb_2_S_3_ are obtained by ALD from the reactions of ZnEt_2_ and Sb(NMe_2_)_3_ with H_2_S, respectively. ZnS serves as tunnel barrier to curb recombination in the photovoltaic device.^25^ The amorphous as-grown sulfide crystallizes to stibnite upon annealing at 300 °C (Fig. 3).^34^

**Fig. 4:**
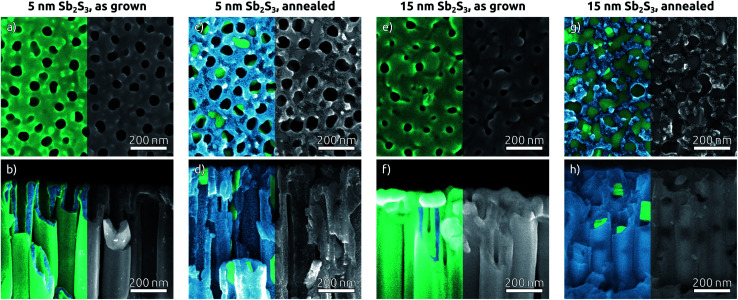
SEM top views (a, c, e and g) and cross sections (b, d, f and h) of TiO_2_ NTs with Sb_2_S_3_ ALD layers before and after annealing with TiO_2_ and Sb_2_S_3_ indicated in blue and green, respectively. (a and b) 15 nm of Sb_2_S_3_ as grown, (c and d) 15 nm of Sb_2_S_3_ crystallized, (e and f) 5 nm of Sb_2_S_3_ as grown, (g and h) 5 nm of Sb_2_S_3_ crystallized.

The morphology of Sb_2_S_3_-coated TiO_2_ NTs is affected by the annealing step, as characterized by SEM (Fig. 4). The top view micrographs of NT arrays reveal a completely smooth surface. The presence of a Sb_2_S_3_ layer is revealed by the narrower NT openings in the case of the thicker coating (see Fig. 4e) (see Fig. 1e for comparison). The cross-section micrographs of the 5 nm and 15 nm thick Sb_2_S_3_ coatings display the expected formation of a conformal layer on both the inner and outer sides of the TiO_2_ NTs (Fig. 4b and f). The conversion of the amorphous ALD solid to stibnite Sb_2_S_3_ is affected by a dewetting effect upon crystallization, depending on the film thickness and morphology of the underlying substrate. On a planar substrate, the ultrathin (1 nm) ZnS interfacial layer is sufficient to avoid dewetting, at least for 15 nm of Sb_2_S_3_ (Fig. S5†).^25^ However, in the highly curved confines of the TiO_2_ NTs, the additional adhesion provided by the ZnS layer is not sufficient to inhibit dewetting completely. Dewetting of the originally conformal 5 nm Sb_2_S_3_ results in the formation of stibnite grains that adhere to the walls of the TiO_2_ NTs but leave the nanochannels open (Fig. 4c and d). What is worse, aggregation of larger Sb_2_S_3_ amounts originating from dewetting a 15 nm layer generates discontinuous rod-like structures shaped by the TiO_2_ nanochannel and sometimes block the open central channel (Fig. 4g and h).

**Fig. 5:**
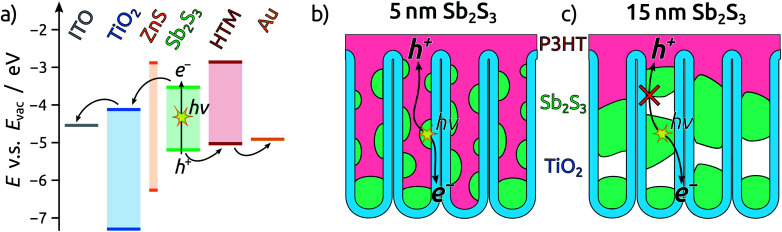
Schematic illustrations of the solar cells prepared in this work. (a) Band diagram of the semiconductor stack. (b and c) Schematic cross-section of a TiO_2_ NT solar cell with 15 nm (b) and 5 nm (c) of Sb_2_S_3_.

To complete the TiO_2_ NT solar cells, P3HT as the hole transport material is infiltrated into the pores by spin coating, followed by electrical contacting with PEDOT:PSS and an evaporated gold electrode. A scheme of the resulting band alignments is depicted in Fig. 5a. The partial withdrawal of Sb_2_S_3_ upon annealing generates direct p–n contacts in addition to the desired p–i–n. This effect is mild in the case of 5 nm Sb_2_S_3_, and it does not cause a clogging of the tube openings so that the infiltration of P3HT is not hindered (Fig. 5b). In contrast to this, 15 nm of Sb_2_S_3_ result in the formation of discontinuous nanorods that block the NT openings and render the lower segments inaccessible to P3HT (Fig. 5c). In this case, hole extraction is interrupted.

### 3.3 Photovoltaic performance

**Fig. 6:**
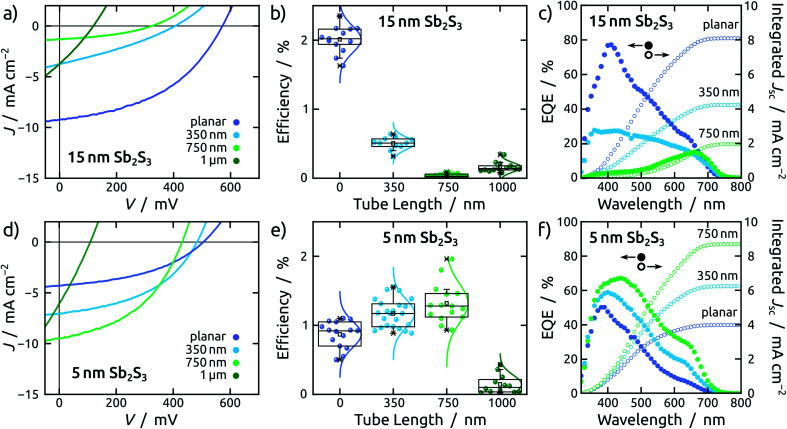
Photovoltaic performance of solar cells based on 15 (a–c) and 5 nm (d–f) of Sb_2_S_3_ on TiO_2_ in planar configuration (purple), and in nanotubes of 350 nm (blue), 750 nm (bright green) and 1 μm (dark green) length. (a–c) *J*–*V* curves, efficiency and EQE with integrated photocurrent density for 15 nm of Sb_2_S_3_. (d–f) Same data collected for 5 nm of Sb_2_S_3_.

This effect transpires in the PV parameters of cells built with the thick light absorbing material (Fig. 4g, h and 5c). Indeed, the series of cells featuring 15 nm of Sb_2_S_3_ exhibits the best performance for the planar configuration (Fig. 6a). The average efficiency of planar solar cells is 2%, while for the NTs it drops abruptly below 0.5% (Fig. 6b). The statistical study of all photovoltaic parameters is in line with this observation (see Fig. S7†). External quantum efficiency spectra (EQE) reveal that charge carriers are generated in the planar device throughout the visible spectrum (Fig. 6c) but suffer from poor extraction. The shift of the EQE maximum from blue to red upon increase of the tube length is a clear indication of it (Fig. 6c and S8a†).

A planar cell with 5 nm Sb_2_S_3_ layer is affected by lower light absorption than with 15 nm, as quantified by the transmittance measurements (see Fig. S6†), therefore it generates a lower current density (Fig. 6d). This 5 nm thick Sb_2_S_3_ layer, however, profits from the interdigitated geometry as expected (Fig. 4c, d and 5b). The large specific surface area of TiO_2_ NTs translates into increased light absorption, as reflected in the transmittance spectra (see Fig. S6c†). This results in a trend whereby longer tubes yield improved performance, until the advantageous effect of increased light absorption is counter-balanced by the deleterious interface recombination of charge carriers, which also increases with pore length (Fig. 6d, e and S8†). This effect becomes predominant for NTs of 1 μm length, which are additionally affected by crack formation in the anodic film (see ESI Fig. S10†).

Therefore, an optimal tube length is found at 750 nm (Fig. 6e), in stark contrast to the devices based on a 15 nm thick Sb_2_S_3_ layer (Fig. 6b). In fact, the champion cell is obtained in a 750 nm NT device and dwarfs the corresponding planar device by a factor close to 2 (Fig. 6e). This increase exclusively originates in the photocurrent, associated with light absorption (*J*_sc_), whereas the open circuit potential (*V*_oc_) decreases with the length of the NTs due to recombination. The fill factor (FF) remains unaffected (see ESI Fig. S8†). EQE spectra exhibit efficient collection of charge carriers generated throughout the visible spectrum for both planar and NT configurations (Fig. 6f).

## 4 Conclusions

In summary, an optimized anodization procedure yields crack-free layers of short (≤1 μm) TiO_2_ nanotubes suitable as a platform for building structured solid-state p–i–n heterojunction. Sb_2_S_3_ is deposited as the absorber material by ALD in high purity and complemented by a polythiophene hole transporter infiltrated from the liquid phase. The straight cylindrical scaffold provided by TiO_2_ NTs translates into a systematically tunable interdigitated device architecture. The generality of this approach is limited by the dewetting effect of Sb_2_S_3_ upon annealing, which affects the morphology and causes carrier recombination at the TiO_2_/P3HT interface. For a so-called “extremely thin absorber” layer of 5 nm, the best devices were obtained with 750 nm long TiO_2_ NTs. These devices double the photocurrent over the reference planar devices by increased light absorption while recombination is maintained at an acceptable level.

This thickness of 5 nm sets a new perspective on the term “extremely thin”. The performance does not reach that of the best planar or mesoporous systems yet, but this model system allows for an outstanding degree of geometric control. We are convinced that using the strategy and preparative methods presented here, significant performance gains can be expected beyond 2% if a satisfying solution to prevent Sb_2_S_3_ dewetting not only on planar surfaces but also concave ones can be found. We are currently exploring avenues towards this goal.

## Conflicts of interest

The authors declare no conflict of interest.

## Supplementary Material

RA-010-D0RA04123H-s001
