# Supplementary material for: Solid state interdigitated Sb2S3 based TiO2 nanotube solar cells
Source: RSC Adv. 2020 Jul 28;10(47):28225–31. doi: 10.1039/d0ra04123h (PMC9055636; doi:10.1039/d0ra04123h)
Supplement: RA-010-D0RA04123H-s001 [file RA-010-D0RA04123H-s001.pdf]

Electronic Supplementary Material (ESI) for RSC Advances.
This journal is © The Royal Society of Chemistry 2020

Solid state interdigitated Sb_2S_3 based TiO_2 nanotube solar cells

Pascal Büttner,[1] Dirk Döhler,[1] Sofia Korenko,[1] Sebastian Möhrlein,[1] Sebastian Bochmann,[1] Nicolas Vogel,[2] Ignacio Mínguez-Bacho,[1, a)] and Julien Bachmann[1,3, b)]

[1)] *Friedrich-Alexander University of Erlangen-Nürnberg, Chemistry of Thin Film Materials, Department of Chemistry and Pharmacy, IZNF, Cauerstr. 3, 91058 Erlangen, Germany.*

[2)] *Friedrich-Alexander University of Erlangen-Nürnberg, Department of Chemical and Biological Engineering, Haberstraße 9a, 91058 Erlangen, Germany.*

[3)] *Saint-Petersburg State University, Institute of Chemistry, Universitetskii pr. 26, 198504 St. Petersburg, Russia.*

(Dated: July 20, 2020)

[a)] Electronic mail: ignacio.minguez@fau.de

[b)] Electronic mail: julien.bachmann@fau.de

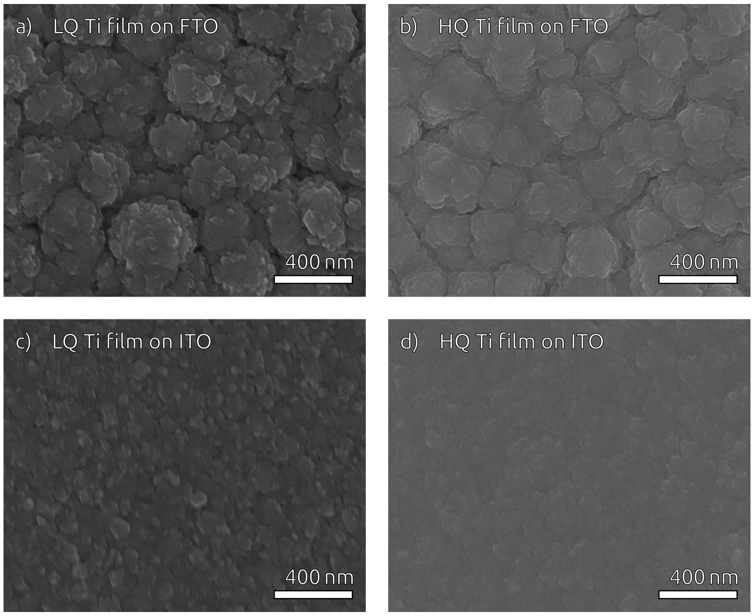

Figure S1. Ti films grown under different conditions on FTO and ITO with a working pressure and power density of 0.3 Pa and 3.3 W cm^{-2} (HQ Ti on ITO) and 0.5 Pa and 2.7 W cm^{-2} (LQ Ti on ITO). a) LQ Ti film grown on FTO, b) HQ Ti film grown on FTO, c) LQ Ti film grown on ITO, d) HQ Ti film grown on ITO.

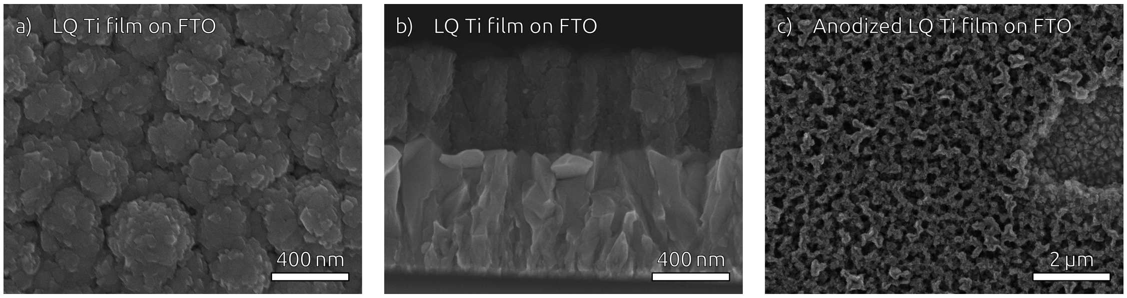

Figure S2. a,b) SEM top view (a) and cross section (b) of LQ Ti films on FTO. c) SEM top view of the anodized LQ Ti film.

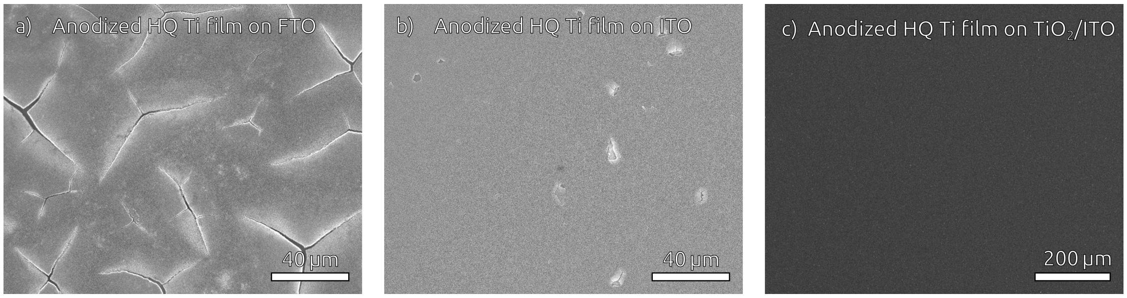

Figure S3. As-anodized HQ Ti films directly grown on FTO (a) and ITO (b) substrates without amorphous TiO_2 blocking layer. c) As-anodized HQ Ti films grown on TiO_2/ITO.

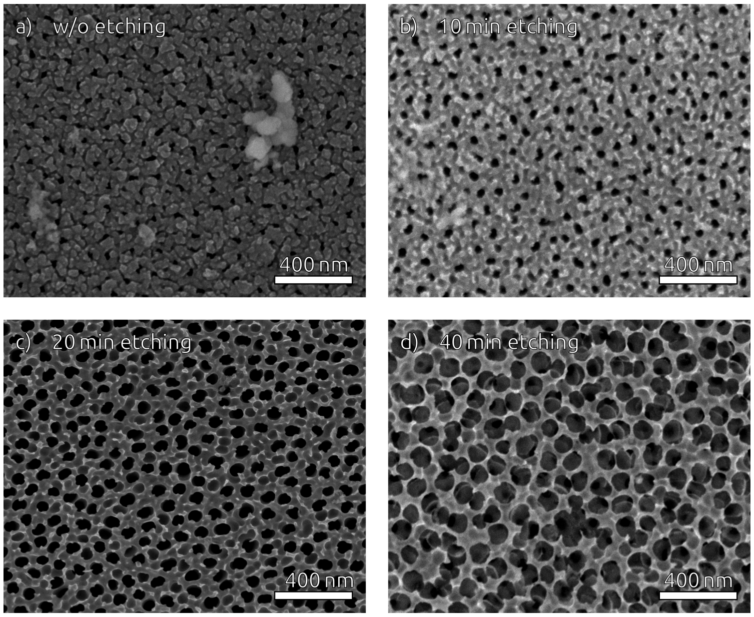

Figure S4. Influence of etching duration on TiO_2 NT morphology. a) without etching, b) 10 min of etching, c) 20 min of etching, d) 40 min of etching.

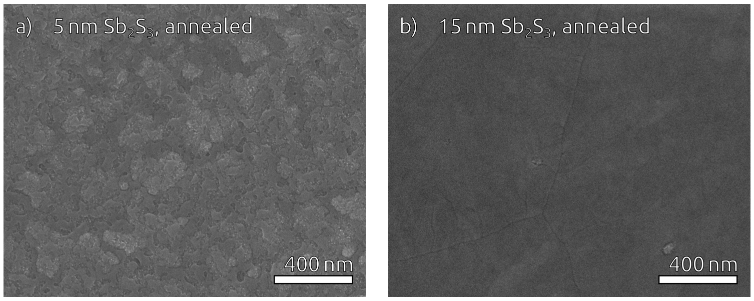

Figure S5. Dewetting effect of 5 nm (a) and 15 nm Sb_2S_3 on planar TiO_2/ITO substrates.

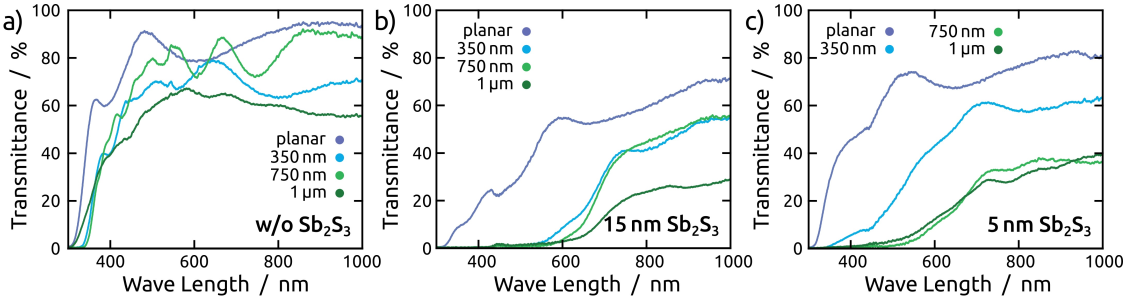

Figure S6. Direct transmission measurements of planar and TiO_2 NT substrates on TiO_2/ITO before (a) and after deposition and crystallization of 15 nm Sb_2S_3 (b) or 5 nm Sb_2S_3.

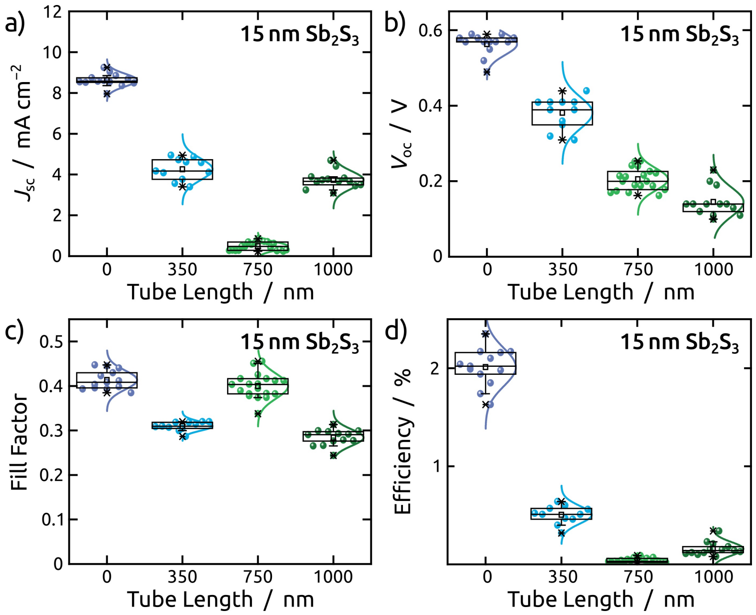

Figure S7. Device statistics for 15 nm of Sb_2S_3 on TiO_2 NT substrates with different NT lengths. a) Short circuit current density, b) open circuit potential, c) fill factor, d) efficiency.

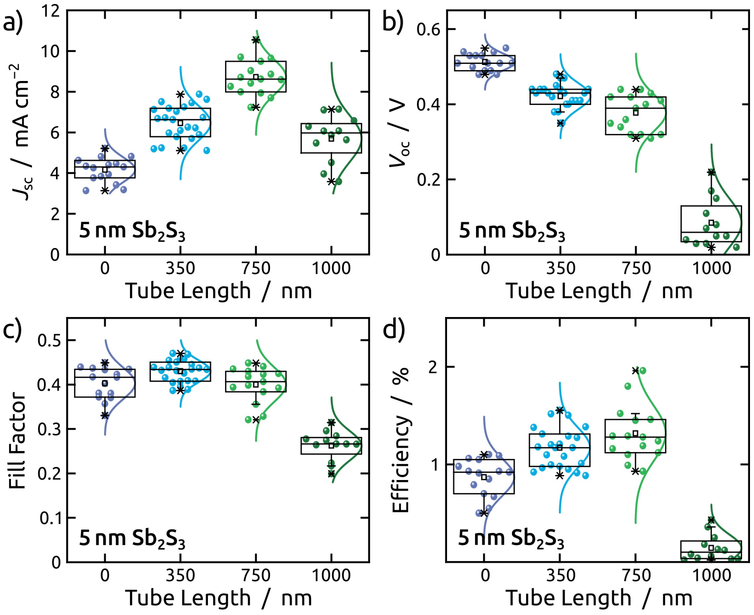

Figure S8. Device statistics for 5 nm of Sb_2S_3 on TiO_2 NT substrates with different NT lengths. a) Short circuit current density, b) open circuit potential, c) fill factor, d) efficiency.

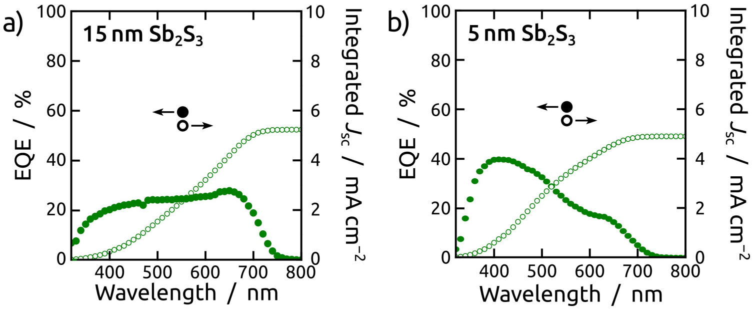

Figure S9. EQE with integrated photocurrent dentsity for TiO_2 NT length of 1 μm with an Sb_2S_3 thickness of a) 15 nm and b) 5 nm.

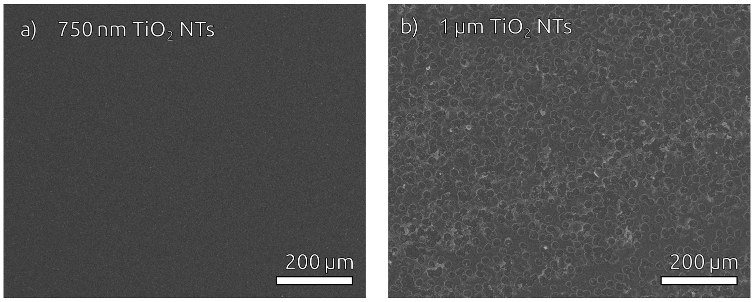

Figure S10. Low magnification SEM images of TiO_2 NT layers for NT lengths of a) 750 nm and b) 1 μm, showing the formation of cracks as the adhesion of the NT layer fails to accommodate the evolving strain due to volume expansion during anodization.

Table S1. Literature summary of Sb_2S_3 solar cells based on well-defined nanostructures and the current record efficiencies for both planar thin film and mesoporous sensitized configuration.

Cell type	Nanostructure	Efficiency [%]	Year	Ref.
Sensitized	mesoporous TiO_2	7.5	2014	[1]
Thin film	planar	6.56	2019	[2]
Thin film	TiO_2 NRs	5.8	2019	[3]
Thin film	TiO_2 NRs	6.78	2018	[4]
Coaxial	Si NRs	0.25	2015	[5]
Coaxial	ZnO NRs	0.2	2019	[6]
Coaxial	ZnO / ZnS NRs	1.32	2014	[7]
Coaxial	TiO_2 NRs	0.4	2019	[6]
Coaxial	TiO_2 NRs	0.67	2016	[8]
Coaxial	TiO_2 NRs	1.47	2013	[9]
Coaxial	TiO_2 NRs	3.76	2019	[10]
Coaxial	TiO_2 NRs	5.37	2020	[11]
Coaxial	TiO_2 dendrides	1.53	2018	[12]
Coaxial	TiO_2 dendrides	1.56	2018	[13]
Coaxial	TiO_2 dendrides	1.83	2019	[14]
Coaxial	TiO_2 NTs	*not specified*	2015	[15]
Coaxial	TiO_2 NTs	0.95	2016	[16]
Coaxial	TiO_2 NTs	2.1	2020	***This work***

REFERENCES

[1]Y. C. Choi, D. U. Lee, J. H. Noh, E. K. Kim and S. I. Seok, *Advanced Functional Materials*, 2014, **24**, 3587–3592.

[2]C. Jiang, R. Tang, X. Wang, H. Ju, G. Chen and T. Chen, *Solar RRL*, 2019, **3**, 1800272.

[3]Y. Yin, C. Wu, R. Tang, C. Jiang, G. Jiang, W. Liu, T. Chen and C. Zhu, *Science Bulletin*, 2019, **64**, 136 – 141.

[4]R. Tang, X. Wang, C. Jiang, S. Li, G. Jiang, S. Yang, C. Zhu and T. Chen, *J. Mater. Chem. A*, 2018, **6**, 16322–16327.

[5]Y.-D. Hsieh, M.-W. Lee and G.-J. Wang, *International Journal of Photoenergy*, 2015.

[6]V. Sharma, T. K. Das, P. Ilaiyaraja and C. Sudakar, *Solar Energy*, 2019, **191**, 400 – 409.

[7]J. Han, Z. Liu, X. Zheng, K. Guo, X. Zhang, T. Hong, B. Wang and J. Liu, *RSC Adv.*, 2014, **4**, 23807–23814.

[8]W. Li, J. Yang, Q. Jiang, Y. Luo, Y. Hou, S. Zhou, Y. Xiao, L. Fu and Z. Zhou, *Journal of Power Sources*, 2016, **307**, 690 – 696.

[9]Y. Li, L. Wei, R. Zhang, Y. Chen, L. Mei and J. Jiao, *Nanoscale Research Letters*, 2013, **8**, year.

[10]C. Ying, C. Shi, K. Lv, C. Ma, F. Guo and H. Fu, *Materials Today Communications*, 2019, **19**, 393 – 395.

[11]C. Ying, F. Guo, Z. Wu, K. Lv and C. Shi, *Energy Technology*, 2020, **8**, 1901368.

[12]Y. Li, Y. Wei, K. Feng, Y. Hao, Y. Zhang, J. Pei and B. Sun, *New J. Chem.*, 2018, **42**, 12754–12761.

[13]Y. Li, Y. Wei, K. Feng, Y. Hao, J. Pei and B. Sun, *Materials Research Express*, 2018, **5**, 065903.

[14]Y. Li, Y. Wei, K. Feng, Y. Hao, J. Pei, Y. Zhang and B. Sun, *Journal of Solid State Chemistry*, 2019, **276**, 278 – 284.

[15]Y. Wu, L. Assaud, C. Kryschi, B. Capon, C. Detavernier, L. Santinacci and J. Bachmann, *J. Mater. Chem. A*, 2015, **3**, 5971–5981.

[16]F. Yang, J. Xi, L.-Y. Gan, Y. Wang, S. Lu, W. Ma, F. Cai, Y. Zhang, C. Cheng and Y. Zhao, *Journal of Colloid and Interface Science*, 2016, **464**, 1 – 9.
